# Medical Jubilee Dinner in Albany

**Published:** 1847-04

**Authors:** 


15. *Medical Jubilee Dinner in Albany.*—On the completion, lately, of fifty years spent in the active practice of medicine, by Dr. William Bay, of Albany, the profession of that city complimented him with a public dinner. The occasion seems to have been an exceedingly agreeable opportunity for the manifestation of those pure and ennobling sentiments which men, devoted to the service of humanity, should exhibit towards each other, and especially towards such as have eminently distinguished themselves by long and laborious devotion to their arduous duties. Prof. T. Romeyn Beck presided, and heightened the festivities by a pleasant sketch of the life of their guest, in connection with historical memoranda of several celebrated members of the profession in New-York, who gave character while they lived, to the science of medicine, and whose names are associated with whatever is great and good, and worthy of imitation by their successors. We were delighted with the account of the doings of the physicians at Albany. In honoring gray hairs, as they did in the person of their excellent neighbor, friend and associate, the venerable Dr. Bay, they will themselves be honored by their medical brethren wherever the circumstances are made know.—*Ibid.*

